# Estimating an Elephant Population Size Through Local Ecological Knowledge

**DOI:** 10.3390/biology13120971

**Published:** 2024-11-25

**Authors:** Michael Wenborn, Magdalena S. Svensson, Vincent Nijman

**Affiliations:** Anthropology and Geography, School of Law and Social Sciences, Oxford Brookes University, Oxford OX3 0BP, UK; 16040486@brookes.ac.uk (M.W.);

**Keywords:** community game guards, elephants, Kunene, local ecological knowledge, Namibia, population estimates

## Abstract

In planning and monitoring measures to protect wildlife in an area, it is important to have a reliable baseline estimate of population size and trends. There has been minimal published information on a small population of elephants, a keystone and endangered species, in a large area west of Etosha National Park in Namibia, known locally as the Northern Highlands. It is a highly remote, mountainous area in which it is difficult to count elephants. It is semi-desert, where the protection of wildlife is at increasing risk from climate change events, and research on the elephant population is a priority. We interviewed 34 community game guards in the Northern Highlands, focusing on the number of elephants and distinguishing features in known groups. Based on the collated knowledge, and analysis to reduce double counting of known groups, we estimate that there are between 78 and 212 elephants in the Northern Highlands, with a best estimate of 128. The wide range is an indication of the current uncertainties in the method. However, we conclude that this low-cost method, if adapted based on lessons from this pilot study, would be applicable for longer-term ecological monitoring in areas that have a low population density.

## Simple Summary

In a mountainous area of northwest Namibia, known as the Northern Highlands, community game guards have many years of experience in monitoring local wildlife. But until our study there had been no published research specifically on the elephants in the Northern Highlands. The area is highly remote, and the steep, rocky landscape makes traditional monitoring methods difficult for this small population of elephants, both in terms of on-the-ground tracking and aerial surveys. We identified that an alternative approach of estimating the size of the elephant population could be to gather local ecological knowledge of game guards. From this method, we estimated that there are between 78 and 212 elephants in the Northern Highlands, with a best estimate of 128. We identified how the method could be improved and conclude that it would be applicable for longer-term ecological monitoring of trends in population. This cost-effective approach would supplement other approaches such as aerial surveys that can only provide a snapshot of the population estimate once every five years, because of the high costs. The long-term monitoring would be important to inform the planning of adaptive strategies for the protection of elephants, an endangered species.

## 1. Introduction

**Figure 1:**
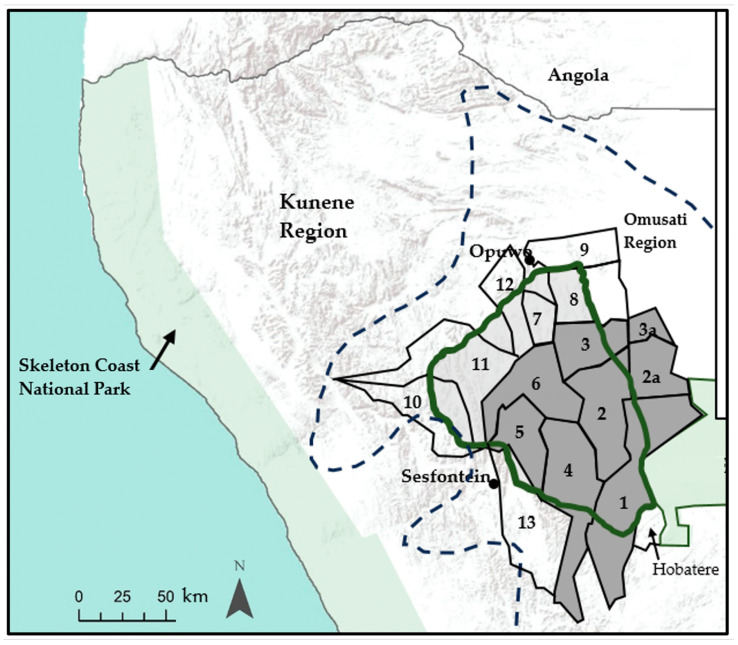
Conservancies in the Northern Highlands of Namibia, highlighting the main six conservancies covered in this study, as well as the elephant range. Key 1: Ehi-Rovipuka; 2: Orupupa; 2a: Additional area monitored by Orupupa; 3: Otuzemba; 3a: Additional area monitored by Otuzemba; 4: Omatendeka; 5: Ozondundu; 6: Okangundumba; 7: Otjombande; 8: Okongoro; 9: Otjindjerese; 10: Otjikondovirongo; 11: Ombujokanguindi; 12: Okatjandja Kozomenje; 13. Anabeb. The thick green line encompasses the Northern Highlands, whereas the conservancies covered in this study are coloured dark grey. According to [1] the range of elephants is within the dashed line.

According to the IUCN African Elephant Database and the most recent IUCN African Elephant Status Report, the African savannah elephants (*Loxodonta africana*) in the Kunene Region, outside of Etosha National Park, are estimated to be about 1.5% of the Namibian elephant population, spread over a very large area of at least 41,000 km^2^ [1,2]. This range includes an area sometimes referred to locally as the Northern Highlands, which is about 12,000 km^2^ (Figure 1). It is a remote, mountainous terrain with steep, rocky slopes and altitudes up to about 1800 m above sea level. Further to the west of the Northern Highlands, the landscape drops, with an escarpment towards the Skeleton Coast.

The Northern Highlands is on the edge of the Namib Desert, which runs along the length of the Namibian coast. The Highlands has low rainfall (typically 50 to 200 mm per year) [3,4]. The rainy season tends to be from January to April. However, some areas receive no rainfall for several years at a time. When it occurs, rainfall tends to be highly localised and intense, in places mobilising large quantities of sediment leading to soil erosion. This process has been exacerbated by anthropogenic activities in terms of over-grazing of livestock in the area [5,6,7], highlighting the dynamic and complex relationship between abiotic and anthropogenic stressors (climate and human activity). Vegetation is sparser just to the west of the Highlands, and game species tend to migrate with the rain to search for fresh grasslands. While much of northwest Namibia is not fit for extensive agricultural development [7,8], livestock farming continues to be the main focus of the community for their livelihoods. Access to grazing and water resources are major priorities in terms of local livelihoods and culture. The potential risk of climate change events is likely to increase the competition between humans (and their livestock) and elephants for natural resources. It is important to have a reliable estimate of the population of elephants as a baseline for monitoring the performance of conservation measures, and to have a cost-effective method to monitor trends in population.

Reliable estimates of elephant numbers in northwest Namibia are difficult to obtain because of the very low density of elephants and the large range covered by the species. In the Northern Highlands, the mountainous terrain and difficult access constrain monitoring activities such as foot patrols and aerial surveys [9,10,11]. The desert-adapted elephants to the west of the Northern Highlands, nearer the Skeleton Coast, have been extensively studied in the lower Hoanib and lower Hoarusib Rivers [12,13,14,15,16,17,18]. These small populations are currently well-known in terms of identification and knowledge of individual groups and some individual elephants, with researchers carrying out almost annual monitoring visits since 2006 and having the benefit of vehicles and equipment (e.g., binoculars, cameras) [17,18]. Until recently, there have been minimal research and publications on the elephants in the Northern Highlands.

Elephants in northwest Namibia often move into the villages to drink at the community water points, and to eat at community vegetable gardens, and there are challenges with human–elephant conflict in northwest Namibia [10,19,20,21], including in the Northern Highlands [22]. The African savannah elephant, a keystone species, is now classified as Endangered in the IUCN Red List of Threatened Species [23]. Namibia’s National Elephant Conservation and Management Plan includes the strategic objectives to plan local interventions to protect the elephants and their habitats in northwest Namibia, including to reduce the incidents of human–elephant conflict [10]. The plan identifies that research is needed to obtain more reliable estimates of the number of elephants in northwest Namibia.

The estimate of the elephant population in northwest Namibia in the most recent IUCN African Elephant Status Report was 314 elephants [1]. This was based on the results of an aerial survey in 2011 of part of the northwest [24], which concluded with a range of elephants with a 95% confidence limit of 221 to 529. An aerial survey in northwest Namibia in 2016 estimated a wide range in the potential elephant population and recommended that, when planning elephant management, a precautionary approach would assume an estimate of 416 elephants in the northwest [25]. These estimates for northwest Namibia compare to the estimated population in Etosha National Park of 2911 elephants [1].

Aerial surveys have indicated that the population of elephants is likely to have been growing in northwest Namibia since the late 1990s [11,24,26]. However, the high costs of the aerial surveys mean that they have not been carried out frequently, and there were only three aerial surveys between 2003 and 2023. Also, there are high uncertainties in the estimated populations from the survey observations [10]. This is mainly because of the very low density of elephants over such a large range area, meaning that it is difficult to scale up sample counts with confidence.

**Figure 2:**
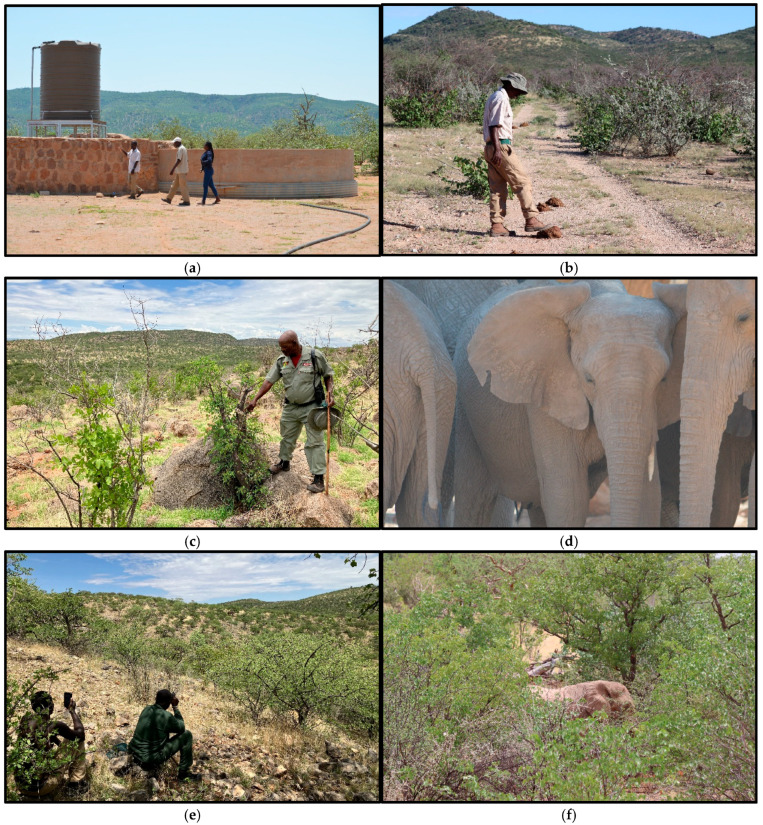
(**a**) Game guard meeting community members to investigate damage by African savannah elephants at a water point in Ekoto, Orupupa Conservancy; (**b**) game guard observing evidence of elephant movements on patrol; (**c**) game guard on patrol identifying a tree partly eaten by elephants as evidence of elephant movements; (**d**) an elephant in the lower Hoanib with a missing tusk and torn ear, as an example of distinguishing features that can help identify individuals or groups of elephants; (**e**) game guards observing an elephant in Ozondundu Conservancy; (**f**) elephant in Omatendeka Conservancy in the shade of trees near the main river bed, demonstrates that it is difficult to observe and count elephants.

The Northern Highlands makes up part of the elephant range in northwest Namibia and most of the area of the Northern Highlands is covered by community conservancies (Figure 1). The conservancy programme has been implemented by the Government of Namibia since the 1990s, then an innovative approach to adapting conservation strategies to the local situation in remote communal areas. One of the benefits of the conservancy programme is the employment of community game guards. Each conservancy tends to employ between five and ten game guards, and some of the game guards have had over 20 years of experience in their roles. They are responsible for recording wildlife sightings and incidents of human–wildlife conflict in Event Books (Figure 2a). Their role also includes raising awareness in communities on the benefits of conservation of habitats and wildlife, in line with the core objectives of the conservancy programme. The game guards have received training from the Ministry of Environment, Forestry and Tourism (MEFT) and the main NGOs involved in community-based wildlife conservation in Namibia (Namibian Association of CBNRM Support Organisations (NACSO), IRDNC Integrated Rural Development and Nature Conservation (IRDNC) and WWF Namibia). They carry out several foot patrols each week, during which they usually do not see elephants, but they will often observe evidence of elephant movements and numbers, such as through footprints, dung and tree species that have been browsed by elephants (Figure 2b,c). They do sometimes observe the elephants when they visit community water points and vegetable gardens, although often that happens at night. The game guards therefore have much useful local ecological knowledge on the elephants in the Northern Highlands, which could inform planning of conservation measures.

The objective of this study was to test an alternative approach to ecological monitoring through estimating the elephant population in the Northern Highlands based on local ecological knowledge, and to assess whether such a method might be applicable in the future for monitoring population trends over the longer term. There have been some positive conclusions on the usefulness of local ecological knowledge from other studies on elephants [27,28].

## 2. Method

This study was carried out by a mix of researchers and practitioners. It involved a total of six months in the Northern Highlands study area, in 2021, 2022 and early 2023. The earlier scoping activities in the study area in 2021 and early 2022 were important to get to know the local situation and plan the method. Scoping activities included consultations with conservancy chairpersons and game guards for ten conservancies. Consultation was also carried out with the Ministry of Environment, Forestry and Tourism to align the research with the objectives and current priorities in Namibia’s National Elephant Conservation and Management Plan [10].

The core research work involved semi-structured interviews. These were carried out over three months (December 2022 to February 2023) with 34 individual game guards in eight conservancies in the Northern Highlands: Ehi-Rovipuka (2 game guards), Orupupa (6), Otuzemba (6), Omatendeka (8), Ozondundu (6), Okangundumba (4), Otjombande (1) and Okongoro (1) (Figure 1). These conservancies were selected because our findings from the scoping activities were that the main conservancies with elephants are in the southern, central and eastern conservancies in the Northern Highlands. The game guards interviewed had between 1 and 30 years of experience, with an average of 10.3 years in their role. The game guards were selected with the agreement from conservancy chairpersons. The game guards interviewed were mainly from the highland areas in the six conservancies. The interviews included all game guards in Orupupa and Ozondundu, all in the highland area of northern Omatendeka, all game guards in Okangundumba except one game guard in the west of the conservancy where elephants are not commonly observed, the core team of game guards in Otuzemba (excluding trainee game guards that had recently been recruited) and two game guards from the more mountainous part of northern Ehi-Rovipuka.

Topics discussed during the semi-structured interviews included the elephant population, elephant movements and potential reasons for their movements, preferred browsing vegetation of elephants and other aspects of elephant behaviour. The most detailed component of the interviews with the game guards involved semi-structured discussion on known groups of elephants (game guards tend to refer to herds of elephants as “groups”). Game guards were asked about their perception of the number of elephants in a known group and any distinguishing features in the group (for example broken tusks, torn ears, etc., of the matriarch or another specific elephant in the group: Figure 2d). Other questions on each group included the main places that they have observed the group; the estimated time spent by the group in the conservancy per year; the number of years that the game guard had been aware of the specific group; and their perception of the typical movements of the group.

The aim was to develop an estimate of the elephant population in the Northern Highlands based on the knowledge of the game guards. For this exercise, we defined a “known group” as a group of elephants that is known by one individual game guard. This means that there would likely be much double counting where many of the 34 game guards interviewed would each know the same group. The questions on the size of group and distinguishing features would help to eliminate double counting by identifying when game guards know the same group. Box 1 provides the part of the semi-structured interview specifically on each known group of elephants.

Box 1. **Questions on each group of elephants known by each game guard**

1. Group reference number: (include name if already named by community)
2. Type of group
  - Female-led group (herd)
  - Informal group of males
  - Individual male
3. Main areas where the group/individual spends time (note if the group is mainly in another conservancy, and note which conservancy)
4. Estimated number of elephants in group (estimate by the game guard)
5. Basis for the estimate (e.g., sightings by game guard, reports from villagers, etc)
6. Distinguishing features of the group that allows it to be identified (e.g., features on matriarch or larger elephants, etc.)
7. How long have you been seeing (and able to identify) the group?
  - Less than 2 years
  - 2 to 5 years
  - More than 5 years
  - Don’t know
8. Overview of movements of elephant group (distance, direction, time of year)
9. Direction of movements in/out of conservancy (if applicable).
10. Amount of time spent outside conservancy per year
11. Perception on reasons for movements of group
12. Problems with human-elephant conflict with this group / individual (High/Medium/Low)
13. Examples of damage caused by herd
14. What is your judgement of the overall uncertainty of the estimated number of elephants in this group? (A, B, C, D, E)
15. Interviewer’s judgement of uncertainty. (A, B, C, D, E)

Many of the times that game guards are close to elephants are at night at village water points, and it is difficult to count the elephants (game guards do not have spotlights, and need to keep a distance for safety reasons: Figure 2e). In the daytime, sometimes elephants are difficult to count when they are spending time in the shade of the taller trees (Figure 2f). In addition, the number of elephants in a group varies, as adult male elephants tend to move in and out of the groups. Game guards know more about some groups than others, some through their own observations and some from feedback about the elephants from community members. Therefore, we developed a simple uncertainty rating and asked game guards to assign a rating to each estimate of number of elephants in each group. The uncertainty ratings for the estimates by game guards of the number of elephants in a group were defined as follows:

- Estimate based on many sightings and observations by the game guard interviewed, verified with much feedback from other game guards and local people, with high confidence.
- Estimate based on some sightings and observations by the game guard interviewed, verified with much feedback from other game guards, and with some confirmation by local people, with good confidence.
- Estimate based on some sightings and observations by the game guard interviewed, some feedback from game guards and local people, with medium confidence.
- Estimate based on a few sightings and observations by the game guard interviewed, some feedback from local people, with low confidence.
- Estimate based on only feedback from local people, no sightings and observations by the game guard interviewed or other game guards, minimal confidence.

## 3. Results

Our approach for the analysis of known groups was to develop an estimate of the elephant population by identifying the likely number of elephant groups that are more permanently in the Northern Highlands and the likely size of each group.

**Figure 3:**
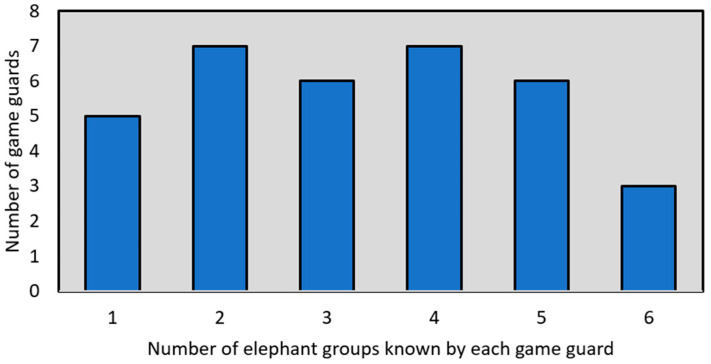
Number of African savannah elephants (*Loxodonta africana*) groups known by game guards in the Northern Highlands of Namibia.

The 34 game guards identified a total of 113 known female-led groups, plus 45 individual males or informal groups of males. Out of the 34 game guards, 16 said that they know four or more female-led groups (Figure 3), but no game guard has knowledge of more than six female-led groups. Individual game guards know 3.3 female-led groups on average.

Our judgement, based on interviews with the game guards and on much time spent in the study area, is that the number of large groups (e.g., of ten or more elephants) is likely to be between three and ten. The 34 game guards in total identified 99 known groups of ten or more elephants, which is an indication of the amount of double counting. Our assumption that there are three to ten larger groups is supported by the results of the aerial survey in 2016, during which four female-led herds of elephants were observed in the Northern Highlands study area [25].

**Figure 4:**
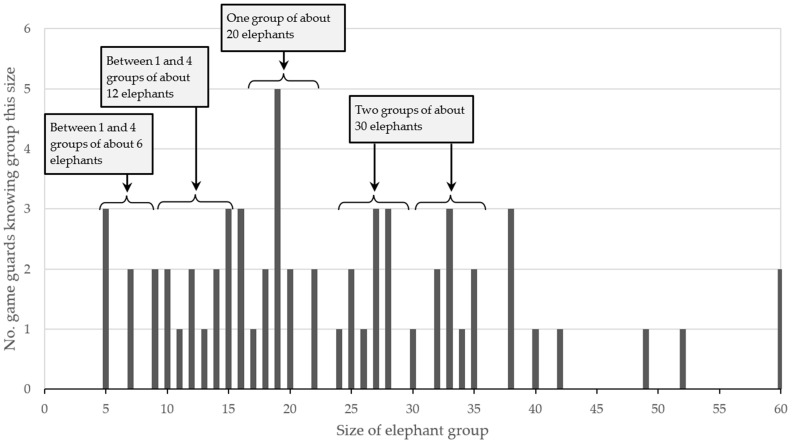
Number of game guards knowing groups of a specific size for the 61 known African savannah elephants (*Loxodonta africana*) groups in Namibia’s Northern Highlands with Category A level of certainty.

For our analysis we focused on the groups that were best known by game guards. Sixty-one known groups were given a Category A rating of uncertainty by game guards (Box 1). Using the plot of these known groups in Figure 4, we divided the set of known groups of Category A into subsets of similar group size. We analysed each subset through visual inspection of the tables of data. For example, we started with a subset of known groups perceived to be containing 16 to 22 elephants. This was because three experienced game guards from Otuzemba Conservancy named a group of 19 elephants that they know (the “Topoti” group). This was the only female-led group to have been given a name, and the game guards have common knowledge of distinguishing features such as a large female elephant in this group with unusually long tusks, one elephant with a torn ear, and one with a large hole in its foot showing in footprints. Splitting the data set of known groups into subsets was useful to eliminate double counting. For example, most of the game guards that know a group of between 16 and 22 elephants only know one group around that size, and we conclude that there is likely to be just one group of about 20 elephants in the Northern Highlands.

Several game guards each know two groups of about 30 elephants (one group is likely to have a little less than 30 elephants and one group a little more (Figure 4)). The different distinguishing features noted by game guards gave some confidence that there are two groups around that size. There had also been reports from game guards on at least two occasions when about 60 elephants have been observed at a water point (e.g., Omunuandjai in Okangundumba Conservancy and Otjitoko in Otuzemba Conservancy), implying that there are two groups of about 30 elephants, which might sometimes visit water points at the same time.

For the elephant herds with lower group sizes, the distinguishing features noted by game guards were not sufficient to make an informed conclusion about the number of groups in the Northern Highlands. Based on the discussions with game guards over the time period in the field, we assumed that there are between one and four groups of around twelve elephants (in a range of nine to fifteen elephants), and between one and four groups of around six elephants (in a range of five to eight elephants).

Adult male elephants often associate in groups. The male elephants tend to keep more distance between them than female-led groups [29,30], and male groups are sometimes observed by game guards when they come together at water points. During the interviews, game guards described several individual male elephants and informal known groups of up to four male elephants. Based on an assessment of the information from game guards on known male elephants during the interviews, we estimate that there are between 10 and 20 adult male elephants moving as individuals or groups in the Northern Highlands. There are uncertainties in this assumption, partly because adult males join female-led groups at times.

From our analysis of the likely number of elephant groups in the Northern Highlands and the likely size of each group, we estimate is that there are between 78 and 212 elephants in the Northern Highlands, with a best estimate of 128 elephants (Table 1). In addition, there are some groups and individuals leaving Etosha National Park for short time periods but spending most of their time in the park, as described by game guards from Ehi-Rovipuka and Orupupa, the conservancies nearest to the park (Figure 1).

## 4. Discussion

### 4.1. Importance of Knowing the Elephant Population

There are increasing stresses to the Northern Highlands habitats and landscape, with the contribution of over-grazing to erosion from high rainfall events, and longer-term droughts also from climate change, both leading to increased conflicts between humans and wildlife for local resources. A reliable estimate of the current number of elephants in the Northern Highlands, and knowledge of the trends in the population, is important as the baseline for adapting conservation strategies and planning measures to protect the elephants, reduce human–elephant conflict and improve safety for the local people. Knowledge to be able to identify individual herds (groups) of elephants, and individual males, would also be useful because common feedback from game guards is that only a proportion of groups and individual males cause incidents with human–elephant conflict. With the coverage of the mobile phone network improving in the area, early warning communication systems would be useful to inform local villages of certain elephants being in the area. Information on specific herds and individual males would also be useful to help plan GPS collaring programmes, which is part of the action plan for the northwest in the Government of Namibia’s National Elephant Conservation and Management Plan.

### 4.2. Contributing to Knowledge on Elephant Movements

One of the interesting points identified from our analysis confirms that known groups are moving long distances all around the Northern Highlands, as concluded in earlier studies using GPS collars in northwest Namibia [31]. This is because the same known groups of about 20 and 30 elephants have been identified by game guards in different locations across several conservancies. For example, the same group of about 19 elephants has been observed in Otjitoko in Otuzemba Conservancy and Okakuyu in Ozondundu Conservancy, which are over 80 km apart on a direct route.

### 4.3. Estimated Population of Elephants

The interviews were carried out in late 2022 and early 2023. The estimated population of 128 elephants (range 78 to 212) can be considered to represent the situation in 2022. Although the known groups identified, on which this estimate is based, were mainly from game guards in six conservancies (highlighted in Figure 1), we consider that this estimate represents the population in the Northern Highlands area in Figure 1. For example, the game guards in Otjombande and Okongoro Conservancies indicated that they observe fewer elephants than those in Otuzemba and Orupupa, and that elephants in those conservancies visit from Otuzemba (and sometimes from Omusati Region in the east) for shorter time periods relative to the groups in the main six conservancies in the study. The indications from wider consultation during our field work were also that elephants in Otjikondovirongo and Ombujokanguindi Conservancies only visit from the southwest for short time periods. Therefore, we consider that including elephants known in those other conservancies would likely be double counting and these elephants are already covered in the above estimate.

### 4.4. Uncertainties in the Estimates of Elephant Populations

There are several uncertainties with the method used to estimate the population from local ecological knowledge on known groups. Based on the interviews and the time spent in the study area, we conclude that there are between three and ten large groups of elephants in the Northern Highlands (e.g., of ten or more elephants in a group). However, the uncertainties relate more to the number of elephants in each group. The game guards tend mostly to see elephants at night, when they are difficult to count. Even in the day, counting of larger groups is difficult because of the vegetation coverage and the fact that game guards need to keep a distance for safety reasons. The number of elephants in a group also often changes, with adult male elephants moving in and out of groups at different times. In particular, the game guards do not have binoculars and spotlights.

The wide range in our estimate from known groups, of 78 to 212 elephants (best estimate 128 elephants), demonstrates the current uncertainties in the method. However, this compares to an estimate of 84 to 325 elephants (best estimate 115 elephants) in the Northern Highlands based on the average elephant density from the reports of two aerial surveys of northwest Namibia [25,26]. It is understood that an aerial survey has been carried out in early 2024. When the results of that survey are available, it would be interesting to compare the estimated elephant population with the findings from this study.

### 4.5. Improving the Method

As would be expected when testing a method, our experience during this study is that the interview questions on groups could be improved, with more precise and detailed questions on distinguishing features. Despite the uncertainties, the method provides low-cost estimates that are based on many years of experience of game guards, compared to high-cost aerial surveys that provide a snapshot estimate once every few years (and there is uncertainty in the estimates from aerial surveys because the elephant density is low). Overall, we conclude that the method could be applied with confidence for long-term ecological monitoring if the game guards were implementing a structured approach over time, using binoculars, spotlights and cameras (with zoom lenses) so that they could more easily identify distinguishing features. Game guards would need to be provided with training. An established NGO, Elephant-Human Relations Aid (EHRA), carries out elephant tracking for the small population of desert-adapted elephants in the Ugab River catchment, over 200 km to the south of the highlands. EHRA has developed an elephant identification protocol [32], which includes a structured approach to identify groups and individual elephants. EHRA trains community game guards in the Ugab area, including in elephant identification methods [33]. Although there are more elephants and more game guards over a much larger area in the Northern Highlands than the Ugab catchment, there is an opportunity for the EHRA training programme to be rolled out in the Northern Highlands, subject to funding, to set up a more structured approach over time, implemented by the community game guards using the EHRA protocol. Improved estimates of the elephant population in the Northern Highlands, from a revised and more structured method that takes into account the lessons from this study and includes statistical analysis, would contribute to planning adaptive measures to protect the elephants, including to reduce human–elephant conflict, at this time when climate change is putting increasing stresses on natural resources in the semi-desert Northern Highlands.

### 4.6. Combining Local Ecological Knowledge with Developing Monitoring Technology

The collation of local ecological knowledge is a growing trend in monitoring species, from specific studies, for example, in Cameroon and Botswana that included local knowledge on elephants [27,28], to wider programmes of citizen science, many of which take advantage of web-based technology on smart phones. The future implementation of approaches discussed above for the game guards in Namibia should keep track of the potential to combine with more modern monitoring techniques that are being researched and developed using technology. Camera traps would also be useful for game guards, but a limitation is the low number of elephants over a large area in the Northern Highlands. The elephants move around to different water points, and their visits to water points that would have camera traps would not be frequent, making identification from the photographs difficult. Monitoring technology using AI has the potential to improve the accuracy and efficiency of studies on elephant populations [34]. AI recognition software attached to camera traps has been tested for elephant monitoring [35,36], but in areas with low elephant population density, such as the Northern Highlands, there would unlikely be a sufficient number of images taken for the technology to identify elephants. Elephants also tend to break camera traps. Satellite technology for counting elephants also has applicability [37], but the low density of elephants in the Northern Highlands, as well as the tree cover along the riverbeds, provide constraints to this approach.

## 5. Conclusions

In planning and monitoring measures to protect wildlife in an area, especially those areas that are remote and least accessible, it is important to have a reliable baseline estimate of population size and trends. Until we initiated our study, there had been minimal published information on the population size, let alone population size changes, of the African savannah elephants in Namibia’s Northern Highlands. The elephants here are a keystone species and form a distinct population of this globally threatened species. Given the aridity of the area, future climate change may disproportionately affect this population, and reliable estimates of current and future population sizes are essential for effective management. The involvement of personnel engaged in conservation is important in testing new methods for monitoring species. We identified that using local ecological knowledge from game guards is an effective method initially to obtain a first estimate of population sizes of these elephants, and secondly that the method could be improved and adapted so that it would be applicable for longer-term ecological monitoring of trends in the population. This cost-effective approach would supplement other approaches such as aerial surveys that cannot be frequently repeated, and can only provide a snapshot of the population, because of the high costs. The long-term monitoring would be important to inform the planning of adaptive strategies for protection of the elephants, as well as other species living at low population densities over vast areas.

## Figures and Tables

**Table 1:** Summary of analysis for estimated number of African savannah elephants (*Loxodonta africana*) in the Northern Highlands study area, based on known groups identified by game guards.

Group Type	Group Size	Range in Number of Groups of This Size	Range in Total Number of Elephants	Best Estimate of Number of Groups	Best Estimate of Number of Elephants
Female-led	30	1–2	30–60	2	60
Female-led	20	1–3	20–60	1	20
Female-led	12	1–4	12–48	2	24
Female-led	6	1–4	6–24	2	12
Individual or groups of males			10–20		12
Estimated total no. of elephants			78–212		128

## Data Availability

The data table of results can be obtained by emailing the corresponding author. Due to sensitivity of the data, the exact locations of the elephants will not be revealed.
